# Investigation into the Influence of Polishing Depth and Speed on the Nano-Polishing Process of Nickel–Phosphorus Alloys via Molecular Dynamics

**DOI:** 10.3390/mi16040444

**Published:** 2025-04-09

**Authors:** Jiadai Xue, Yutao Liu, Qiuyan Liao, Ziteng Li, Fei Ding, Yuan Jin, Duo Li, Yanwen Liu, Chuanrui Zhu, Yangong Wu, Bo Wang

**Affiliations:** 1 Center for Precision Engineering, Harbin Institute of Technology, Harbin 150001, China; 15765517664@163.com (J.X.); liuyutao_001@126.com (Y.L.); 13654653927@163.com (Q.L.); 22s108282@stu.hit.edu.cn (Z.L.); fei.ding@hit.edu.cn (F.D.); jinyuan@hit.edu.cn (Y.J.); bradywang@hit.edu.cn (B.W.); 2 State Key Laboratory of Mechanical System and Vibration, School of Mechanical Engineering, Shanghai Jiao Tong University, Shanghai 200240, China; duo.li@sjtu.edu.cn; 3 College of Mechanical and Electrical Engineering, Northeast Forestry University, Harbin 150040, China; liuyw@nefu.edu.cn; 4 Beijing Changfeng Kewei Photoelectric Technology Co., Ltd., Beijing 100854, China; zhu.chuanrui@foxmail.com

**Keywords:** nickel–phosphorus, nano-polishing, molecular dynamics methodology, surface generation, material removal

## Abstract

Nickel–phosphorus (NiP) alloys have been widely used in many engineering fields such as aerospace, automotive, and optics; however, it is difficult to study the material removal mechanism and microscopic size changes in the polishing process of nickel–phosphorus alloys through simple experiments. In light of these difficulties, there is a need to improve our understanding of the surface friction and wear mechanisms of NiP materials. In the present study, molecular dynamics simulations are employed for the first time to investigate the material removal mechanism, mechanical response, phase transformation, and stress distribution of two NiP alloys with different phosphorus contents during the nano-polishing process by adjusting the polishing depth and speed. Our simulation results indicate that the mechanical response of the low-phosphorus alloy is slightly higher than that of the high-phosphorus NiP alloy. Larger polishing depths and higher speeds reduce the surface quality and lead to increased residual stress. The findings presented herein provide an atomic-level understanding of the material removal mechanism of NiP alloys via MD methodology and offer valuable guidance for selecting alloys with an appropriate NiP ratio as engineering materials and for developing processing methods to improve surface quality.

## 1. Introduction

Nickel–phosphorus (NiP) is an alloy prepared through electroplating or electroless plating techniques that possesses excellent corrosion resistance [1,2,3], high hardness [4,5], and superior mechanical properties [6,7,8] combined with good machinability [9,10]. It plays a significant role in the fields of aerospace, automotive, and optical engineering [11,12,13]. Studying the mechanical properties of NiP alloys can therefore aid in better understanding their characteristics, thereby facilitating the exploration of broader application fields for such alloys. Nano-machining technology, as an important ultra-precision machining technique, can efficiently process materials with high shape accuracy and excellent surface roughness, meeting the demands for NiP surface quality in various fields, particularly in terms of reducing roughness on optical surfaces [14,15,16].

Bao et al. [17] conducted orthogonal ultra-precision turning experiments to investigate the influence of cutting parameters on the quality of nickel–phosphorus alloys. They found that increasing the cutting speed can improve the surface quality of nickel–phosphorus alloys and reduce the cutting force. Negative rake angle tools increase the cutting force but result in a smoother surface; in comparison, positive rake angle tools increase the surface roughness. Pramanik et al. [18] performed ultra-precision cutting on electroless nickel-plated mold materials. They found that an increase in cutting distance leads to an increase in surface roughness, with an increase in feed rate significantly increasing surface roughness. Moreover, spindle speed within a certain range reduces surface roughness; however, excessively high spindle speeds can degrade surface quality. In addition, an increase in the phosphorus content of the alloy reduces surface roughness. Xu et al. [19] analyzed the factors affecting polishing and studied the surface quality improvement methods for NiP coatings on metal mirrors, determining the optimal polishing slurry formula and processing parameters. Dong et al. [20] experimentally compared the differences between diamond fly cutting and transverse planing for nickel–phosphorus machining. They found that diamond fly cutting is a more effective method for machining micro-pyramid arrays on nickel–phosphorus coatings, offering higher machining efficiency and better quality compared to transverse planing. Yang et al. [21] studied the isotropic etching polishing (IEP) of NiP coating surfaces through experiments and verified that NiP surfaces with better surface quality can be obtained through IEP. Although experimental studies have revealed the effects of cutting parameters on surface roughness and cutting forces, they fail to capture atomic-scale phenomena such as subsurface phase transformations, stress evolution, and material removal mechanisms. Additionally, the role of phosphorus content in dynamic mechanical responses during polishing remains unexplored.

During the nanoscale machining of NiP alloys, the subsurface and internal microstructure of the alloy will undergo alteration; however, it is difficult to observe the internal changes in the alloy during the machining process through experimental research. Molecular dynamics (MD) simulation is an effective method to observe material behavior at the nanoscale, including dislocation propagation, amorphous-to-crystalline phase transitions, and stress distribution patterns that are inaccessible through conventional characterization techniques [22,23,24]; concurrently, with the improvement of computer performance, MD can aid in the rapid comprehension of the processing process at the atomic level and aid in the avoidance of the adverse effects induced by other factors during experiments [25]. At present, a number of scholars have used the MD method to study the nano-processing process of materials [26,27,28]. Yin et al. [29] studied the deformation mechanism of the 3C-SiC polishing process with a rotating abrasive through molecular dynamics simulations, with the results showing that the normal force increases first and then decreases with the increase in abrasive rotational speed. Liu et al. [30] used the MD method to simulate the process of nano-polishing 3C-SiC and investigated the influence of polishing speed on the material removal mechanism. They hypothesize that the increase in polishing speed will reduce surface quality and the lifespan of the abrasive but that it can also suppress phase transformation in the subsurface. Hadipour et al. [31] investigated the wear behavior of gradient NiP coatings with a medium phosphorus top layer (Ni-MP) through experiments and molecular dynamics simulations. They found that the Ni-MP top layer possessed higher hardness, a compact surface morphology, and a gradient crystal structure. The wear resistance of the nickel–low-phosphorus (Ni-LP)/Ni-MP bilayer was 1.5 times higher than that of the single Ni-MP layer. During the wear process, the gradient structure suppressed crack propagation, with it being concluded that gradient Ni-P coatings could significantly improve the wear resistance of materials. Li et al. [32] used molecular dynamics simulations to study the nanoscale cutting mechanisms of high-phosphorus NiP coatings under different cooling rates. They found that with the increase in cooling rate, the degree of amorphization, atomic disorder, and the number of atoms on the workpiece surface all increased; however, the actual machined surface profile deviated from the ideal surface profile. Despite these advancements, our understanding of the changes in the subsurface during the NiP polishing process remains insufficient; as such, there is an urgent need to study the influence of polishing parameters on the subsurface through molecular dynamics research.

In this study, we used molecular dynamics simulations to analyze the material removal mechanism, phase transformation, mechanical response, and stress variations in two NiP alloys with different phosphorus contents during nano-polishing processes under different speeds and depths. The research results showed that the phosphorus content has a significant impact on the properties of the material and the polishing parameters play a crucial role in forming a high-quality surface. The findings provide theoretical support and valuable guidance on atomic mechanisms for the ultra-precision manufacturing of NiP alloys.

## 2. Methods

**Figure 1:**
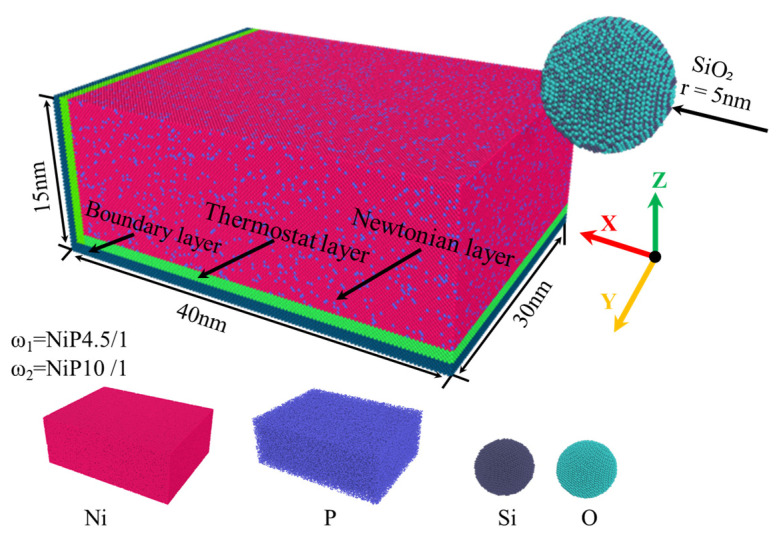
MD model of the SiO₂ abrasive nano-polishing amorphous NiP alloy.

To investigate the influence of polishing depth and speed on the nano-polishing process of NiP alloys, a molecular dynamics model of the nano-polishing process was established, as shown in Figure 1, with the model including a NiP alloy sample and a silica abrasive. The silica abrasive is a sphere with a diameter of 10 nm, containing a total of 22,792 atoms, of which the gray atoms are silicon atoms and the cyan atoms are oxygen atoms. The abrasive is located in the upper right corner of the sample. The sample is a rectangular prism with dimensions of 40 nm × 30 nm × 15 nm, containing a total of 1,640,075 atoms. In the sample, the red atoms are nickel atoms and the purple atoms are phosphorus atoms. The NiP alloy sample is divided into three layers from top to bottom: the Newton layer, the thermostat layer, and the fixed layer. The atoms in the Newton layer follow Newton’s second law of motion [33]. The atoms in the thermostat layer follow the Berendsen thermal law, and their temperature remains constant throughout the entire nano-polishing process [34]. The atoms in the fixed layer remain stationary to maintain the stability of the entire nano-polishing system [35]. During the nano-polishing process, the silica abrasive is treated as a rigid body to ensure that the relative positions of the abrasive atoms remain unchanged during the working process. The nano-polishing depth is controlled by changing the vertical distance between the abrasive and the upper surface of the sample. The movement direction of the abrasive is the X direction, and the polishing plane is parallel to the upper surface of the sample. The X and Z directions are free boundary conditions, and the Y direction is a periodic boundary condition. The entire simulation process was conducted using LAMMPS software (64-bit 15Jun2023-MPI) [36].

Before conducting the nano-polishing simulation, it is necessary to prepare amorphous NiP alloys with the appropriate Ni and P contents. In this simulation, the parameters of MD are shown in Table 1; the method of high-temperature melting was employed, followed by cooling to prepare low-phosphorus and high-phosphorus NiP alloys. A total of 1,640,075 atoms were melted at a high temperature of 2000 K. The low-phosphorus and high-phosphorus alloys contained 1,490,993 and 1,341,880 Ni atoms, respectively, and 149,082 and 298,195 P atoms, respectively. After being kept at high temperature for 1 nm, the alloys were quenched to obtain amorphous NiP alloys [37]. Once this process was complete, the high-phosphorus NiP alloy ω_1_ = NiP4.5/1 and the low-phosphorus NiP alloy ω_2_ = NiP10/1 were obtained. The nano-polishing process is divided into three stages. The first stage is the relaxation stage before polishing, during which the entire system is relaxed to minimize its energy. After relaxation, the system is relaxed for 100 ps in the constant volume and temperature (NVT) ensemble. The second stage is the nano-polishing stage, which is conducted in the constant volume and energy (NVE) ensemble. The last stage is the relaxation stage after polishing, in which the system is relaxed in the NVT ensemble to stabilize the atoms in the sample. Once the nano-polishing process is complete, Ovito is used to extract and visualize the MD results to study the atomic changes in the material [38].

The accuracy of MD simulations depends on the precision of the interatomic potential function. The EAM potential can explain the behavior of metal atoms surrounded by other atoms and is an effective potential function for describing the interaction between Ni and P atoms in NiP alloys [39], and the accuracy of the potential energy function was also verified through Scanning Electron Microscope (SEM) experiments [40]. The expression is shown in Equation (1) as follows:

(1) $$\begin{array}{l} U=\sum_{i}E_{i}(\rho_{i})+\frac{1}{2}\sum_{i}\sum_{j\neq i}\varphi_{ij}(r_{ij})\\ \rho_{i}=\sum_{j\neq i}^{i}\rho_{j}(r_{ij}) \end{array},$$

where *E_i_* is the embedding energy of particles, *φ_ij_* is the repulsive force between particles, *ρ_i_* is the sum of the densities produced by the extranuclear electrons of all particles except particle *i* acting on particle *i*, *ρ_j_* is the density produced by the extranuclear electrons of particle *j* acting on particle *i*, and *r_ij_* is the distance between particle *i* and particle *j*.

## 3. Results and Discussion

**Figure 2:**
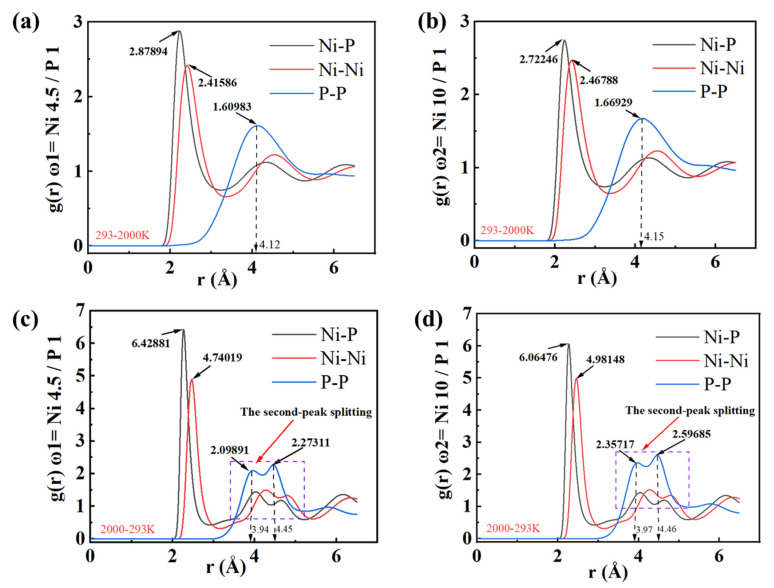
Radial distribution function after melting and cooling of the workpiece. (**a**) ω_1_ = NiP4.5/1 after high-temperature melting; (**b**) ω_2_ = NiP10/1 after high-temperature melting; (**c**) ω_1_ = NiP4.5/1 after cooling; (**d**) ω_2_ = NiP10/1 after cooling.

The radial distribution function (RDF) of atoms is an effective method for analyzing the phase transformation process of materials [41], and it represents the probability of finding an atom at a given distance from the center atom and is a dimensionless parameter. The results presented in Figure 2 show the RDF curves of two NiP alloys with different phosphorus contents during the nano-polishing process as the temperature rises and falls. The RDF curve of NiP exhibits three main peaks, corresponding to the covalent bond lengths of Ni-P, Ni-Ni, and P-P. During the polishing process, as the material’s temperature rises, as shown in Figure 2a,b, it can be observed that the height of the Ni-P bond peak in the high-phosphorus NiP alloy is higher than that in the low-phosphorus NiP alloy. This finding indicates that the number of Ni-P covalent bonds is greater in the high-phosphorus NiP alloy. The peak heights of the P-P and Ni-Ni bonds are similar to that of the Ni-P bond, suggesting that there are fewer Ni-Ni and P-P bonds in the high-phosphorus NiP alloy. After polishing, when the alloy cools, the peak heights of all bonds increase, as shown in Figure 2c,d, and the P-P covalent bond exhibits two peaks. These results reflect the characteristics of amorphous alloys, which lack long-range order. The structure is disrupted during polishing, increasing atomic disorder and resulting in higher peak intensities. In addition, it can be observed that the high-phosphorus NiP alloy experiences greater structural damage compared to the low-phosphorus NiP alloy.

**Figure 3:**
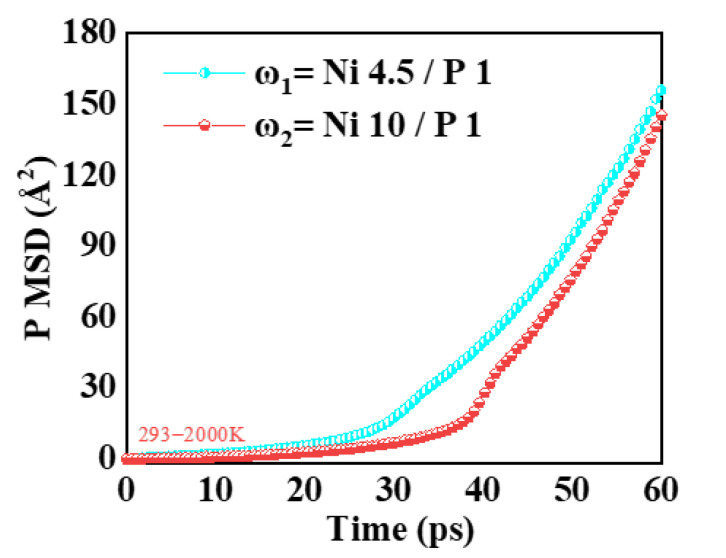
Mean squared displacement curves of P atoms within the amorphous NiP alloy in the Ni workpiece at 293–2000 K.

The mean square displacement (MSD) curve is a method for describing the displacement of atoms in a material over time and is an effective tool for studying the microscopic dynamic behavior within materials. Figure 3 shows the MSD curves for two NiP alloys with different phosphorus contents as the temperature increases during the polishing process. As nano-polishing progresses and the surface temperature of the sample rises, as shown in Figure 3, the MSD curve of the high-phosphorus NiP alloy is higher than that of the low-phosphorus NiP alloy. This indicates that as the phosphorus content decreases, the interactions between particles in the sample strengthen, leading to an increase in disorder. Moreover, as the temperature rises, particle movement within the NiP alloy samples becomes more pronounced, and their interactions become more significant.

**Figure 4:**
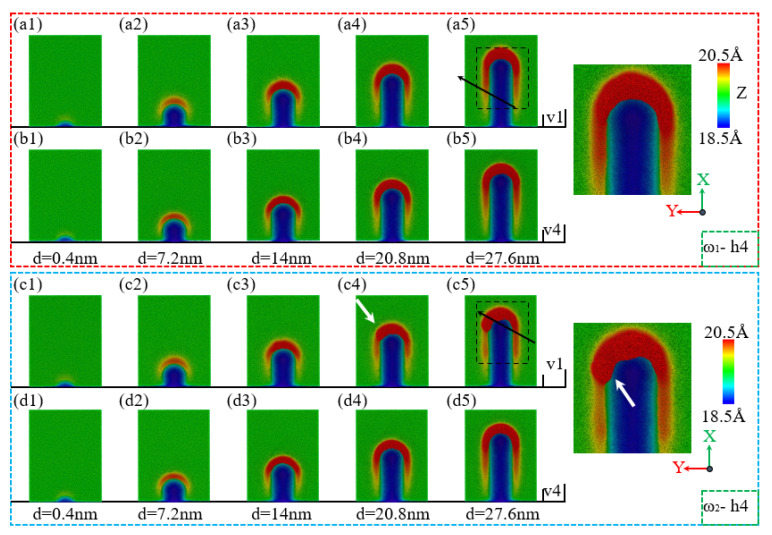
Surface morphology changes of ω_1_ high-phosphorus and ω_2_ low-phosphorus alloys at a polishing depth of h = 4 nm with polishing speeds of v = 1 m/s and v = 4 m/s as a function of polishing distance d. (**a1**–**a5**,**b1**–**b5**) Surface morphology changes of ω_1_-h4 at polishing speeds of v = 1 m/s and v = 4 m/s, respectively. (**c1**–**c5**,**d1**–**d5**) Surface morphology changes of ω_2_-h4 at polishing speeds of v = 1 m/s and v = 4 m/s, respectively.

During the nano-polishing of amorphous NiP alloys using silica abrasives, the surface morphology of the NiP alloy is influenced by the phosphorus content in the material. As shown in Figure 4(a1–a5,b1–b5), for ω_1_ = NiP4.5/1, the accumulation range and height of surface material increase with the polishing distance during the nano-polishing process. However, when comparing the polishing speeds of v = 1 m/s and v = 4 m/s, it is evident that the material accumulation on the surface is almost identical under both speeds, indicating that polishing speed has little impact on material accumulation for high-phosphorus NiP alloys. In contrast, for ω_2_ = NiP10/1, significant differences were observed at a polishing speed of v = 1 m/s. As shown in Figure 4(c1–c5), the material accumulation in front of the abrasive gradually increases with polishing distance [41]. However, when the polishing distance d exceeds 20.8 nm, the material accumulation on the left side of the workpiece along the X direction decreases, and the accumulation shifts toward the left of the abrasive. This shifted accumulation becomes most pronounced at a polishing distance of 27.6 nm. In addition, the local region on the right-hand side of the images has been magnified to enable better observation. For the low-phosphorus NiP alloy ω_2_ = NiP10/1, the surface material accumulation under different polishing distances is generally consistent with the accumulation behavior of the high-phosphorus NiP alloy ω_1_ = NiP4.5/1 at a polishing speed of v = 4 m/s. These findings suggest that polishing speed has minimal influence on the surface morphology of NiP alloys, whereas phosphorus content has a more significant impact on the surface morphology.

**Figure 5:**
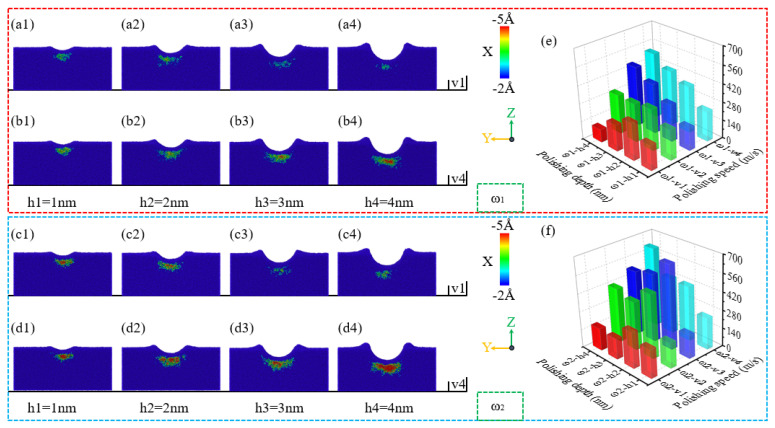
Effect of different polishing depths on material accumulation at the sample entry during the nano-polishing process of ω_1_ high-phosphorus and ω_2_ low-phosphorus NiP alloys at polishing speeds of v = 1 m/s and v = 4 m/s. (**a1**–**a4**,**c1**–**c4**) Correspondence to ω_1_ high-phosphorus and ω_2_ low-phosphorus NiP alloys, respectively, at a polishing speed of v = 1 m/s. (**b1**–**b4**,**d1**–**d4**) Correspondence to ω_1_ high-phosphorus and ω_2_ low-phosphorus NiP alloys, respectively, at a polishing speed of v = 4 m/s. (**e**,**f**) Bar charts of the number of accumulated atoms at the sample entry for ω_1_ and ω_2_ under different polishing conditions.

During the nano-polishing process of NiP alloys using silica abrasives, material accumulation at the sample entry is influenced by the phosphorus content in the alloy, in addition to the polishing depth and speed. As shown in Figure 5(a1), for the ω_1_ high-phosphorus NiP alloy, relatively low amounts of material accumulate at the abrasive entry at a polishing speed of v = 1 m/s and a polishing depth of h = 1 nm. With an increase in polishing depth, as shown in Figure 5(a2–a4), at h = 2 nm, the accumulation range and height increase at the abrasive entry. However, as the depth further increases to h ≥ 3 nm, the height and range of material accumulation at the abrasive entry gradually decrease with depth. At a polishing speed of v = 4 m/s, as shown in Figure 5(b1–b4), the material accumulation at the abrasive entry for the high-phosphorus NiP alloy exhibits a consistent increase. With increasing polishing depth, both the height and range of material accumulation progressively grow; this is consistent with previous research findings [42].

In general, as illustrated in Figure 5(a1–a4,b1–b4,c1–c4,d1–d4), the trends in material accumulation range and height at the abrasive entry are similar for both polishing speeds, v = 1 m/s and v = 4 m/s. However, it is noteworthy that under varying phosphorus content, the ω_2_ low-phosphorus NiP alloy exhibits a significantly greater material accumulation range and height at the abrasive entry compared to the ω_1_ high-phosphorus NiP alloy. In addition, in Figure 5e,f, bar charts are presented in XXY format that statistically compare the number of accumulated atoms at the sample entry for ω_1_ and ω_2_ under different polishing conditions. These visual representations provide a clearer and more intuitive explanation of the material accumulation differences between the high-phosphorus and low-phosphorus amorphous NiP alloys.

**Figure 6:**
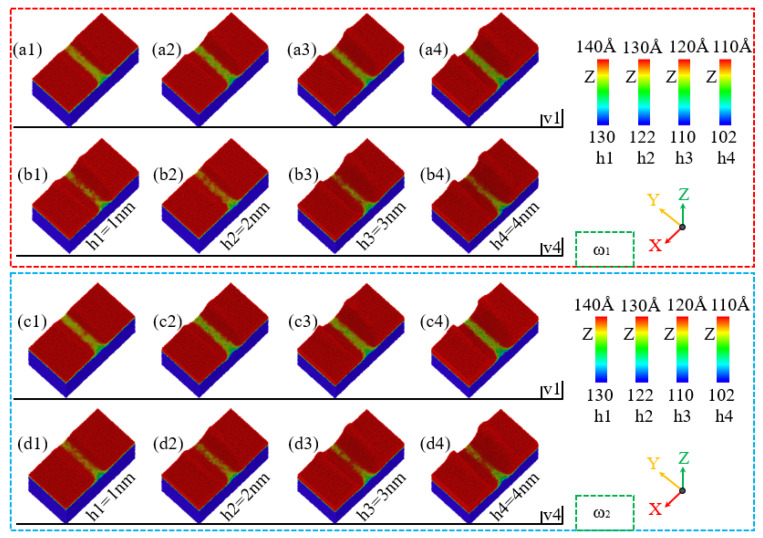
Effects of different polishing depths on sample entry collapse and polishing groove formation during the polishing process of NiP alloys with ω_1_ high-phosphorus and ω_2_ low-phosphorus at polishing speeds of v = 1 m/s and v = 4 m/s. (**a1**–**a4**,**c1**–**c4**) Sample entry collapse and groove morphology for ω_1_ high-phosphorus and ω_2_ low-phosphorus alloys, respectively, at a polishing speed of v = 1 m/s. (**b1**–**b4**,**d1**–**d4**) Corresponding effects for ω_1_ high-phosphorus and ω_2_ low-phosphorus alloys, respectively, at a polishing speed of v = 4 m/s.

During the nano-polishing process of amorphous NiP alloys, sample collapse occurs at the abrasive entry, and the extent of collapse is influenced by polishing speed and depth. As shown in Figure 6(a1–a4,c1–c4), when the polishing speed is v = 1 m/s, both high-phosphorus and low-phosphorus samples exhibit collapse at the entry. The collapsed region increases with polishing depth, and the entry collapse for samples with different phosphorus contents is nearly identical. In addition, as the polishing depth increases, the groove width on the polished sample surface also increases. As illustrated in Figure 6(a1–a4,b1–b4,c1–c4,d1–d4), the entry collapse for samples with different phosphorus contents remains almost identical at the same polishing depth. However, as the polishing speed increases, the entry collapse range at the same polishing depth widens and the flatness of the grooves decreases this result is similar to the research of Komanduri et al. [43].

**Figure 7:**
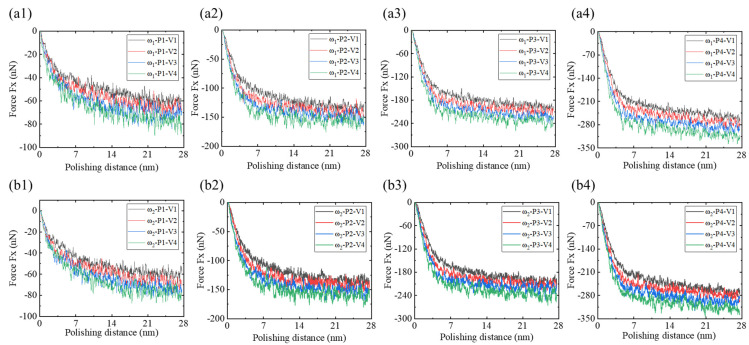
Polishing force Fx for ω_1_ high-phosphorus and ω_2_ low-phosphorus alloys at polishing depths of 1 nm, 2 nm, 3 nm, and 4 nm under polishing speeds of v = 1 m/s, v = 2 m/s, v = 3 m/s, and v = 4 m/s. (**a1**–**a4**) Polishing force Fx for the ω_1_ high-phosphorus alloy at polishing depths of 1 nm, 2 nm, 3 nm, and 4 nm. (**b1**–**b4**) Polishing force Fx for the ω_2_ low-phosphorus alloy at polishing depths of 1 nm, 2 nm, 3 nm, and 4 nm.

During the nano-polishing process of amorphous NiP alloys, the strong interactions between the abrasive and the workpiece generate polishing forces that can be decomposed into tangential force Fx, orthogonal force Fy, and normal force Fz [44]. Figure 7 presents the tangential force Fx variation curves under different polishing depths and speeds for NiP alloys with differing phosphorus contents. As shown in Figure 7(a1–a4,b1–b4), the tangential force Fx acting on both ω_1_ high-phosphorus and ω_2_ low-phosphorus samples increases progressively with polishing distance and is consistent with previous research findings [17]. For polishing distances d ≤ 9 nm, Fx increases rapidly; in comparison, for d > 9 nm, the growth rate of Fx slows, showing a more gradual increase with increasing polishing distance. In addition, as the polishing speed increases, the tangential force Fx acting on both high-phosphorus and low-phosphorus NiP alloys increases. Comparing the Fx behavior of ω_1_ and ω_2_ alloys under various polishing conditions reveals that the fluctuation amplitude of Fx for the ω_2_ low-phosphorus alloy is slightly higher than that of the ω_1_ high-phosphorus alloy at a speed of v = 1 m/s. For speeds v ≥ 2 m/s, the overall magnitude of Fx for the ω_2_ low-phosphorus alloy is slightly higher than that of the ω_1_ high-phosphorus alloy.

**Figure 8:**
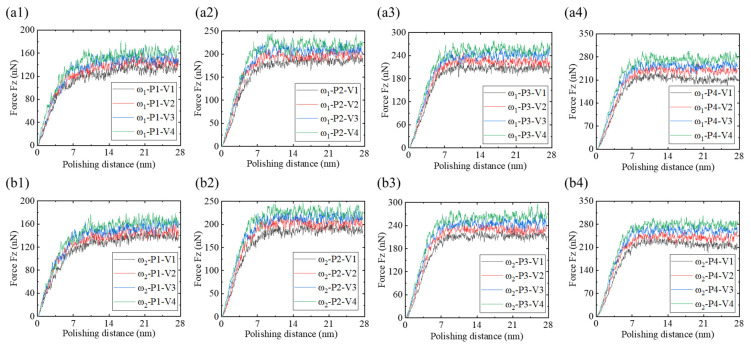
Normal force Fz for ω_1_ high-phosphorus and ω_2_ low-phosphorus NiP alloys at polishing depths of 1 nm, 2 nm, 3 nm, and 4 nm under polishing speeds of v = 1 m/s, v = 2 m/s, v = 3 m/s, and v = 4 m/s. (**a1**–**a4**) Normal force Fz for the ω_1_ high-phosphorus alloy at polishing depths of 1 nm, 2 nm, 3 nm, and 4 nm. (**b1**–**b4**) Normal force Fz for the ω_2_ low-phosphorus alloy at polishing depths of 1 nm, 2 nm, 3 nm, and 4 nm.

Figure 8 illustrates the variation curves of normal force Fz with respect to nano-polishing depth and speed for ω_1_ high-phosphorus and ω_2_ low-phosphorus amorphous NiP alloys during nano-polishing. As shown in Figure 8(a1–a4,b1–b4), the normal force Fz acting on both ω_1_ and ω_2_ samples gradually increases with polishing distance. When the polishing distance d ≤ 9 nm, Fz increases rapidly. However, for d > 9 nm, the increase rate of Fz slows, although it continues to rise gradually with further increases in polishing distance. Moreover, as the polishing speed increases, the normal force Fz on both ω_1_ and ω_2_ samples also increases. In addition, with increasing polishing depth, the increase in Fz is consistent for the two alloys with different phosphorus contents. However, the amplitude of Fz fluctuations for the ω_2_ low-phosphorus alloy is generally slightly higher than that of the ω_1_ high-phosphorus alloy at speeds of v = 1 m/s, 2 m/s, 3 m/s, and 4 m/s.

**Figure 9:**
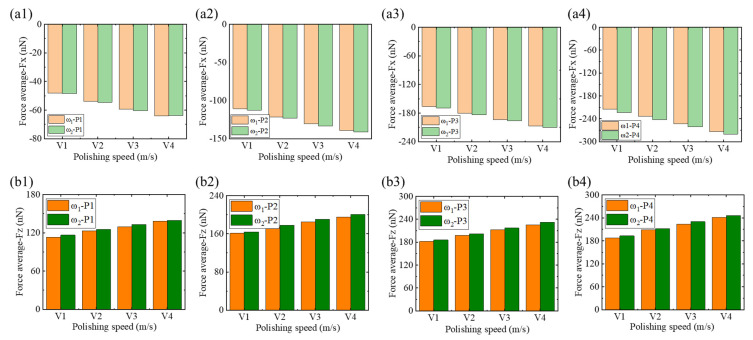
Bar charts of the average tangential force Fx and normal force Fz for ω_1_ high-phosphorus and ω_2_ low-phosphorus alloys at polishing depths of 1 nm, 2 nm, 3 nm, and 4 nm under polishing speeds of v = 1 m/, v = 2 m/s, v = 3 m/s, and v = 4 m/s. (**a1**–**a4**) Average tangential force Fx for the ω_1_ high-phosphorus alloy. (**b1**–**b4**) Average normal force Fz for the ω_2_ low-phosphorus alloy.

In Figure 9, the average values of Fx and Fz acting on ω_1_ high-phosphorus and ω_2_ low-phosphorus alloys under different polishing speeds at the same polishing depth are shown. As shown in Figure 9(a1–a4,b1–b4), the average values of Fx and Fz increase with the polishing speed and polishing depth. It can also be seen that, compared to ω_1_, the average force acting on the ω_2_ low-phosphorus alloy is greater. As shown in Figure 9(a1,b1), when the polishing depth is 1 nm and the polishing speeds are 1, 2, and 3 m/s, the average values of Fx and Fz acting on the ω_1_ high-phosphorus alloy are lower than those acting on the ω_2_ low-phosphorus alloy. However, when the polishing speed is 4 m/s, the average Fx acting on ω_1_ increases to a value greater than that acting on ω_2_, whereas Fz remains lower. The average value of Fx on ω_1_ reaches −70 nN, compared to −68 nN for ω_2_. When the polishing speed is 4 m/s and the polishing depth is 4 nm, the Fx and Fz acting on ω_1_ reach maximum values of 274 nN and 240 nN, respectively; in comparison, those acting on ω_2_ reach maximum values of 280 nN and 250 nN, respectively. The above findings indicate that during the nano-polishing process of NiP alloys, an increase in polishing depth and speed leads to an increase in the average force, and the increase in phosphorus content in the alloy also results in higher average forces.

**Figure 10:**
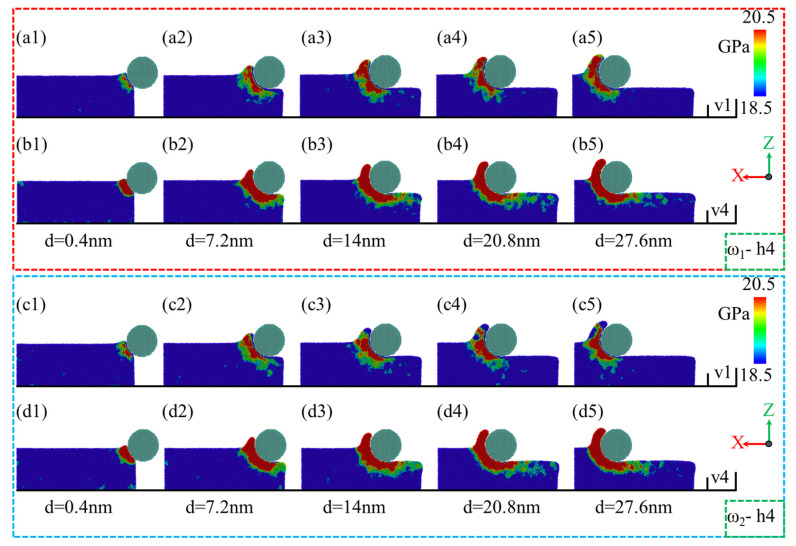
The influence of polishing distance variation on the stress distribution in the XZ cross-section of the sample for ω_1_ high-phosphorus and ω_2_ low-phosphorus alloys under polishing speeds of v = 1 m/s and v = 4 m/s. (**a1**–**a5**,**b1**–**b5**) Stress distribution for ω_1_ high-phosphorus alloy. (**c1**–**c5**,**d1**–**d5**) Stress distribution for ω_2_ low-phosphorus alloy.

During the nano-polishing process of amorphous NiP alloys, the structure of nickel and phosphorus atoms on the sample surface and subsurface undergoes change, resulting in the generation of residual stress. The results presented in Figure 10 illustrate the impact of a polishing depth of h = 4 nm, different speeds, and polishing distances on the internal stress distribution within the XZ cross-section of ω_1_ high-phosphorus and ω_2_ low-phosphorus NiP alloy specimens. As the polishing distance increases, as shown in Figure 10(a1–a5,b1–b5), for the ω_1_ high-phosphorus NiP alloy, the range and magnitude of internal stress distribution within the XZ cross-section gradually increase during nano-polishing. The stress is mainly concentrated in the area in front of and below the silica abrasive. It can also be observed that at higher polishing speeds, the stress in front of the abrasive becomes greater and more uniform, and more residual stress appears in the subsurface after polishing.

As shown in Figure 10(c1–c5,d1–d5), for the ω_2_ low-phosphorus specimen, when the speed v = 1 m/s, the high-stress region in the XZ cross-section is primarily concentrated below the abrasive, whereas the stress in front is lower. At v = 4 m/s, residual stress is generated in the subsurface after polishing, with stress mainly concentrated in front of and below the abrasive. Under different polishing conditions, the high-stress region and the distribution depth and range of residual stress within the XZ cross-section of the ω_2_ low-phosphorus alloy are significantly higher than those of the ω_1_ high-phosphorus alloy.

**Figure 11:**
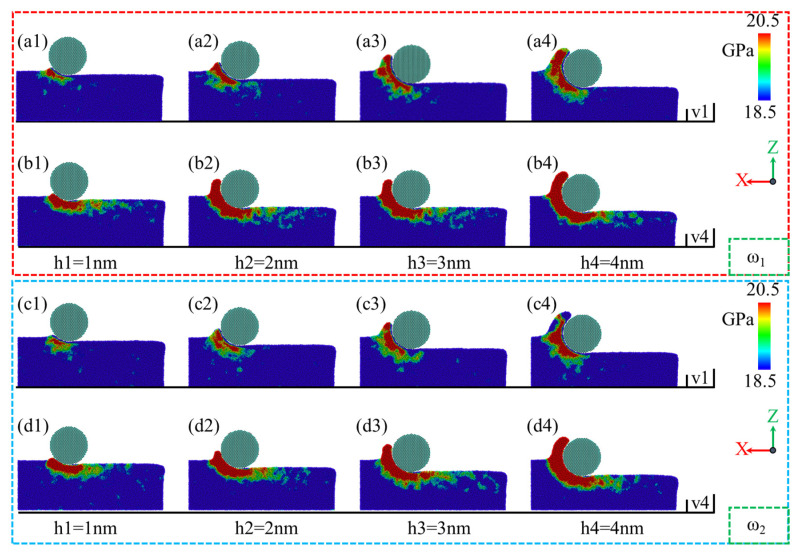
The stress distribution in the XZ cross-section for ω_1_ high-phosphorus and ω_2_ low-phosphorus alloys at different polishing depths under polishing speeds of v = 1 m/s and v = 4 m/s. (**a1**–**a4**,**b1**–**b4**) Stress distribution for the ω_1_ high-phosphorus alloy. (**c1**–**c4**,**d1**–**d4**) Stress distribution for the ω_2_ low-phosphorus alloy.

Figure 11 illustrates the variation in the distribution range of internal stress regions within the XZ cross-section for ω_1_ high-phosphorus and ω_2_ low-phosphorus alloys at different polishing depths at a polishing distance of d = 27.6 nm and polishing speeds of v = 1 m/s and v = 4 m/s. For the high-phosphorus NiP alloy, as shown in Figure 11(a1–a5,b1–b5), under different polishing depths, the distribution range and depth of high-stress regions within the XZ cross-section at v = 4 m/s are significantly greater than those at v = 1 m/s. The stress is mainly concentrated below the abrasive and increases with the polishing depth. In the subsurface layer of the polished sample, residual stress is not observed in the XZ cross-section at v = 1 m/s; in comparison, considerable residual stress appears at v = 4 m/s. For the low-phosphorus NiP alloy, as shown in Figure 11(c1–c5,d1–d5), the trends in the distribution of high-stress regions and residual stress within the XZ cross-section are generally consistent for both polishing speeds, v = 1 m/s and v = 4 m/s. Comparing the two alloy specimens at different polishing depths and speeds, the low-phosphorus alloy exhibits a noticeably greater distribution range and depth of high-stress regions and residual stress within the XZ cross-section compared to the high-phosphorus alloy.

**Figure 12:**
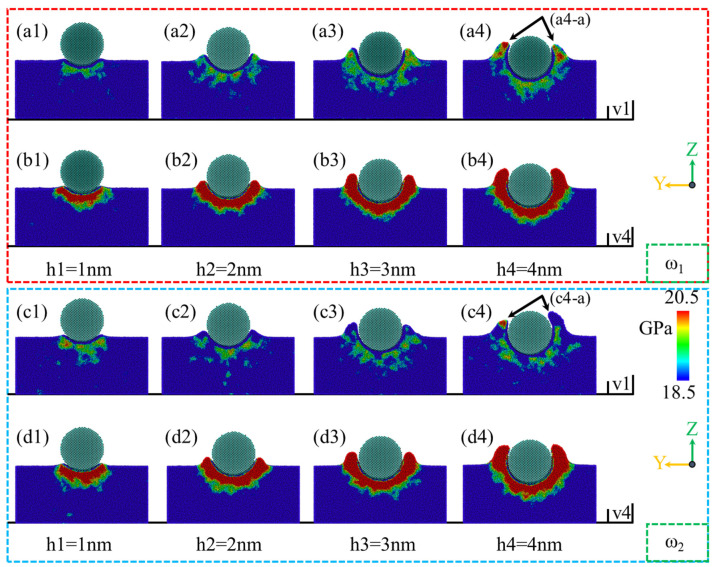
The stress distribution in the YZ cross-section for ω_1_ high-phosphorus and ω_2_ low-phosphorus alloys at a polishing distance of d = 27.6 nm, under polishing speeds of v = 1 m/s and v = 4 m/s, and with varying polishing depths. (**a1**–**a4**,**b1**–**b4**) Stress distribution for the ω_1_ high-phosphorus alloy. (**c1**–**c4**,**d1**–**d4**) Stress distribution for the ω_2_ low-phosphorus alloy.

Figure 12 illustrates the variation in internal stress distribution within the YZ cross-section for amorphous NiP alloys with different phosphorus contents during silica abrasive nano-polishing at a polishing distance of d = 27.6 nm, under varying polishing speeds and depths. As shown in Figure 12(a1–a4,c1–c4), at a polishing speed of v = 1 m/s, the distribution range and depth of internal stress regions within the YZ cross-section significantly increase with increasing polishing depth for both ω_1_ and ω_2_ samples. The stress regions are primarily concentrated below and at the sides of the abrasive. When the polishing depth is h = 1 nm, as shown in Figure 12(a1,b1,c1,d1), increasing the polishing speed leads to higher stress below the abrasive and larger stress concentration areas. With the same polishing speed, the internal stress of the ω_1_ high-phosphorus sample is smaller than that of the ω_2_ low-phosphorus sample. With increasing polishing depth, differences in the internal stress distribution within the YZ cross-section of samples with different phosphorus contents emerge. As shown in Figure 12(a1–a4,b1–b4,c1–c4,d1–d4), when the polishing depth exceeds h = 1 nm, the internal stress of the ω_1_ high-phosphorus sample increases and becomes more widely distributed than that of the ω_2_ low-phosphorus sample. In addition, the stress magnitude increases with increasing polishing speed. At a polishing depth of h = 4 nm, the stress at the sides of the abrasive surpasses that below the abrasive.

**Figure 13:**
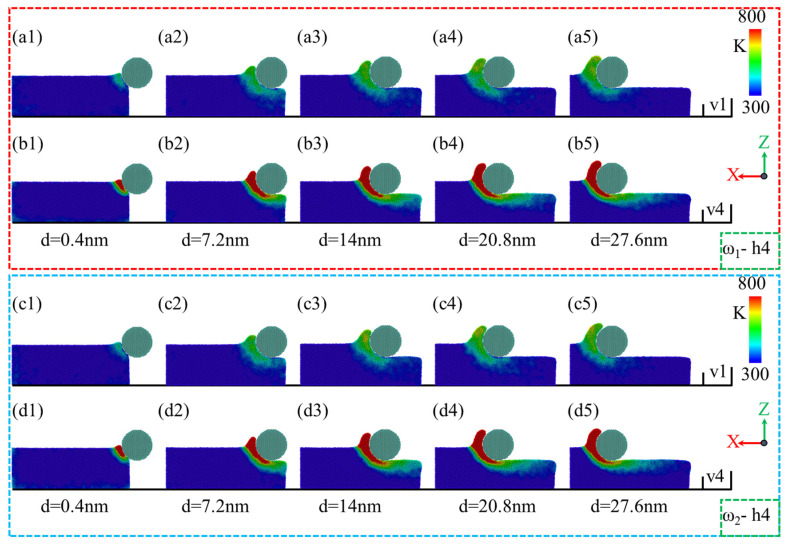
The influence of polishing distance on the temperature distribution within the samples for ω_1_ high-phosphorus and ω_2_ low-phosphorus alloys under polishing speeds of v = 1 m/s and v = 4 m/s. (**a1**–**a5**,**b1**–**b5**) Temperature distribution at different polishing distances for the ω_1_ high-phosphorus alloy. (**c1**–**c5**,**d1**–**d5**) Temperature distribution at different polishing distances for the ω_2_ low-phosphorus alloy.

During the polishing process, the strong interaction between the abrasive and the alloy leads to a rise in temperature within the contact region. The results displayed in Figure 13 show the internal temperature distribution of the XZ cross-section for NiP alloy samples with different phosphorus contents during silica abrasive nano-polishing under varying polishing distances and speeds. As shown in Figure 13(a1–a5,b1–b5), for high-phosphorus NiP alloys, the temperature in the regions in front of and below the abrasive gradually increases with increasing polishing distance. At a polishing speed of v = 1 m/s, the temperature does not exceed 500 K, and the subsurface temperature of the polished area rapidly returns to the initial temperature. However, at a polishing speed of v = 4 m/s, the temperature in the regions in front of and below the abrasive reaches 800 K, with a more intense interaction between the abrasive and the alloy. This factor results in a wider area of elevated temperature and slower recovery of the subsurface temperature post-polishing. For low-phosphorus NiP alloys, as depicted in Figure 13(c1–c5,d1–d5), a similar trend is observed. The internal temperature of the XZ cross-section increases with polishing distance, and the temperature at v = 4 m/s is significantly higher and more widely distributed than that at v = 1 m/s. In addition, a noticeable residual temperature distribution remains in the subsurface grooves after polishing. Compared to the ω_1_ high-phosphorus alloy, both the internal temperature and residual temperature distribution of the low-phosphorus NiP alloy are higher.

## 4. Conclusions

In conclusion, the phosphorus content, polishing depth, and speed play critical roles in the nanoscale polishing of amorphous NiP alloys. The above findings provide theoretical guidance and practical insights for optimizing the polishing process. In this work, we investigated the effects of nanoscale polishing with silica abrasives on amorphous NiP alloys through molecular dynamics simulations, focusing on the influence of phosphorus content, polishing depth, and speed on material removal mechanisms, surface morphology, residual stress distribution, and temperature variation. The results show that the phosphorus content significantly impacts polishing performance as follows:

1. Low-phosphorus alloys exhibit larger material accumulation areas and larger heights at the abrasive entry point, and their surface morphology is more sensitive to the polishing depth and speed, with greater susceptibility to surface collapse. As the polishing depth and speed increase, tangential and normal forces also rise, with low-phosphorus alloys showing slightly higher mechanical responses compared to high-phosphorus alloys.
2. Residual stress, primarily located beneath and in front of the abrasives, expands in range and depth at higher polishing speeds, with low-phosphorus alloys exhibiting a wider and deeper stress distribution.
3. In terms of temperature, the intense interaction between abrasives and alloys results in an increase in the temperature in the contact region. Higher polishing speeds result in more significant temperature increases, with low-phosphorus alloys showing a greater rise.

## Figures and Tables

**Table 1:** Nano-polishing of NiP alloys—MD model parameters.

Parameter	Value
Workpiece Material	ω_1_ = NiP4.5/1 and ω_2_ = NiP10/1
Tool Material	Silica
Atomic Number	ω_1_: Ni = 1,341,880 *p* = 298,195; ω_2_: Ni = 1,490,993 *p* = 149,082; Tool: 47,239
Workpiece Dimensions	400 Å × 300 Å × 150 Å
Tool Shape	Spherical, radius 50 Å
Boundary Conditions	pps (periodic boundary condition in the Y direction; free boundary conditions in the X and Z directions)
Simulation Software	LAMMPS 64-bit 15Jun2023-MPI
Potential Function	Eam/alloy Tersoff
Timestep	1 fs (0.001 ps)
Polishing Temperature	293 K
Polishing Distance	27.6 nm
Polishing Depth	1, 2, 3, and 4 nm
Polishing Speed	1, 2, 3, and 4 m/s

## Data Availability

Data are contained within the article.

## References

[1] Lee C.K. (2009). Structure, electrochemical and wear-corrosion properties of electroless nickel-phosphorus deposition on CFRP composites. Mater. Chem. Phys..

[2] Yang Q., Peng X., Hu H. (2022). Study on optimization of a NiP layer polishing process on magnetorheological finishing for metal substrate. Proceedings of the 7th Asia Pacific Conference on Optics Manufacture.

[3] Kobayashi R., Xu S., Shimada K., Mizutani M., Kuriyagawa T. (2017). Defining the effects of cutting parameters on burr formation and minimization in ultra-precision grooving of amorphous alloy. Precis. Eng..

[4] Wang G., Zhou T., Guo W., Yao X., Yu Q. (2024). Surface modification of nickel-phosphorus plating by Ti ion implantation for chalcogenide glass lens molding. Surf. Interfaces.

[5] Zhou T., Yan J., Liang Z., Wang X., Kobayashi R., Kuriyagawa T. (2015). Development of polycrystalline Ni-P mold by heat treatment for glass microgroove forming. Precis. Eng..

[6] Schuh C.A., Hufnagel T.C., Ramamurty U. (2007). Mechanical behavior of amorphous alloys. Acta Mater..

[7] Inoue A. (2000). Stabilization of Metallic Supercooled Liquid and Bulk Amorphous Alloys. Acta Mater..

[8] Yan J., Oowada T., Zhou T., Kuriyagawa T. (2009). Precision machining of microstructures on electroless-plated NiP surface for molding glass components. J. Mater. Process Technol..

[9] Wang W.H. (2007). Roles of minor additions in formation and properties of bulk metallic glasses. Prog. Mater. Sci..

[10] Xu C., Peng X., Hu H., Liu J., Li H., Luo T., Lai T. (2023). Nano-Precision Processing of NiP Coating by Magnetorheological Finishing. Nanomaterials.

[11] Mainier F.B., Fonseca M.P.C., Tavares S.S.M., Pardal J.M. (2013). Quality of Electroless Ni-P (Nickel-Phosphorus) Coatings Applied in Oil Production Equipment with Salinity. J. Mater. Sci. Chem. Eng..

[12] Kocabaş M., Örnek C., Curioni M., Cansever N. (2019). Nickel fluoride as a surface activation agent for electroless nickel coating of anodized AA1050 aluminum alloy. Surf. Coat. Technol..

[13] Xu Z., Su Y., Yang H., Guo R., Liu X., Zhang F., Liu X., Ji T., Zhang Y., Yuan Y. (2019). Optical processing on the φ150mm SiCp/Al coating nickel phosphorus alloy plane mirror. Proceedings of the Ninth International Symposium on Advanced Optical Manufacturing and Testing Technologies.

[14] Jain V.K., Sidpara A., Sankar M.R., Das M. (2012). Nano-finishing techniques: A review. Proc. Inst. Mech. Eng. Part C J. Mech. Eng. Sci..

[15] Kinast J., Beier M., Gebhardt A., Risse S., Tünnermann A. (2015). Polishability of thin electrolytic and electroless NiP layers. Proceedings of the Conference Optifab 2015.

[16] Wu B., Zong W. (2022). A modified diamond micro chiseling method for machining large scale retroreflective microstructure on nickel phosphorus alloy. J. Mater. Process. Technol..

[17] Bao X., Yao P., Xu J., Mei Z., Li Y., Yang J., Wang Q., Chen Z., Qu S., Huang C. (2023). Effect of tool geometry and cutting parameters on surface quality and chip morphology of amorphous electroless nickel-phosphorus alloy in ultra-precision turning. Int. J. Adv. Manuf. Technol..

[18] Pramanik A., Neo K.S., Rahman M., Li X.P., Sawa M., Maeda Y. (2003). Cutting performance of diamond tools during ultra-precision turning of electroless-nickel plated die materials. J. Mater. Process. Technol..

[19] Xu C., Peng X., Liu J., Hu H., Lai T., Yang Q., Xiong Y. (2022). A High Efficiency and Precision Smoothing Polishing Method for NiP Coating of Metal Mirror. Micromachines.

[20] Dong X., Zhou T., Pang S., Liang Z., Yu Q., Ruan B., Wang X. (2019). Comparison of fly cutting and transverse planing for micropyramid array machining on nickel phosphorus plating. Int. J. Adv. Manuf. Technol..

[21] Yang J., Ye J., Liu G., Ye Z., Cui W., Zhang X., Deng H. (2024). High efficiency polishing of micro-structured NiP alloy using isotropic electrochemical etching for achieving sub-nanometer roughness. J. Manuf. Process..

[22] Xu Y., Wang M., Zhu F., Liu X., Chen Q., Hu J., Lu Z., Zeng P., Liu Y. (2019). A molecular dynamic study of nano-grinding of a monocrystalline copper-silicon substrate. Appl. Surf. Sci..

[23] Goel S., Luo X., Agrawal A., Reuben R.L. (2015). Diamond machining of silicon: A review of advances in molecular dynamics simulation. Int. J. Mach. Tools Manuf..

[24] Feichtinger D., Derlet P.M., Van Swygenhoven H. (2003). Atomistic simulations of spherical indentations in nanocrystalline gold. Phys. Rev. B.

[25] Tranchida D., Piccarolo S., Deblieck R.A.C. (2006). Some experimental issues of AFM tip blind estimation: The effect of noise and resolution. Meas Science and Technology.

[26] Sharma A., Datta D., Balasubramaniam R. (2018). Molecular dynamics simulation to investigate the orientation effects on nanoscale cutting of single crystal copper. Comput. Mater. Sci..

[27] Kim D.E., Oh S.I. (2006). Atomistic simulation of structural phase transformations in monocrystalline silicon induced by nanoindentation. Nanotechnology.

[28] Zhang J., Zhang J., Wang Z., Hartmaier A., Yan Y., Sun T. (2017). Interaction between phase transformations and dislocations at incipient plasticity of monocrystalline silicon under nanoindentation. Comput. Mater. Sci..

[29] Yin Z., Zhu P., Li B., Xu Y., Li R. (2021). Atomic Simulations of Deformation Mechanism of 3C-SiC Polishing Process with a Rolling Abrasive. Tribol. Lett..

[30] Liu H., Zhao P., Wu D., Li D., Wang S., Gao X., Wang D., Wu X., Huang S., Tan J. (2024). Investigate on material removal of 3C-SiC crystals in nano-polishing via molecular dynamics. J. Manuf. Process..

[31] Hadipour A., Daneshmand H., Monirvaghefi S.M. (2024). Wear behavior of graded nickel-phosphorus coatings with a medium phosphorus top layer: An experimental and molecular dynamics simulation study. Colloids Surf. A Physicochem. Eng. Asp..

[32] Li H., Peng X., Guan C., Hu H. (2023). Molecular dynamics simulation of the nano-cutting mechanism of a high-phosphorus NiP coating. J. Mater. Res. Technol..

[33] Wang Y., Tang S., Guo J. (2020). Molecular dynamics study on deformation behaviour of monocrystalline GaN during nano abrasive machining. Appl. Surf. Sci..

[34] Chen M., Dai H. (2022). Molecular dynamics study on grinding mechanism of polycrystalline silicon carbide. Diam. Relat. Mater..

[35] Khan H.M., Kim S.G. (2011). On the wear mechanism of thin nickel film during AFM-based scratching process using molecular dynamics. J. Mech. Sci. Technol..

[36] Plimpton S. (1995). Fast Parallel Algorithms for Short-Range Molecular Dynamics. J. Comput. Phys..

[37] Sadeghilaridjani M., Yang Y.C., Hasannaeimi V., Mahajan C., Jha S., Pole M., Xia Z., Mukherjee S. (2021). Multiscale manufacturing of amorphous alloys by a facile electrodeposition approach and their property dependence on the local atomic order. ACS Appl. Mater. Interfaces.

[38] Stukowski A. (2010). Visualization and analysis of atomistic simulation data with OVITO-the Open Visualization Tool. Model. Simul. Mater. Sci. Eng..

[39] Sheng H.W., Ma E., Kramer M.J. (2012). Relating dynamic properties to atomic structure in metallic glasses. JOM.

[40] Zhang Z., Xu H., Zhou X., Guo T., Pang X., Volinsky A.A. (2021). Deformation Mechanisms of NiP/Ni Composite Coatings on Ductile Substrates. Coatings.

[41] Li C., Piao Y., Meng B., Zhang Y., Li L., Zhang F. (2022). Anisotropy Dependence of Material Removal and Deformation Mechanisms during Nanoscratch of Gallium Nitride Single Crystals on (0001) Plane. Appl. Surf. Sci..

[42] Li Z., Kang S., Liu H., Liu Y., Ren M., Zhang X., Zhu L., Li D. (2024). Molecular Dynamics Study on Burr Formation Mechanism during Monocrystalline Silicon Nano-Grinding Process. J. Manuf. Process..

[43] Komanduri R., Chandrasekaran N., Raff L.M. (2001). MD Simulation of Exit Failure in Nanometric Cutting. Mater. Sci. Eng. A.

[44] Li C., Zhang F., Meng B., Liu L., Rao X. (2017). Material removal mechanism and grinding force modelling of ultrasonic vibration assisted grinding for SiC ceramics. Ceram. Int..

